# The analysis of densitometric and geometric parameters of bilateral proximal phalanges in horses with the use of peripheral quantitative computed tompgraphy

**DOI:** 10.1186/1751-0147-54-41

**Published:** 2012-07-13

**Authors:** Małgorzata Dzierzęcka, Anna Charuta

**Affiliations:** 1 Department of Morphological Science, Faculty of Veterinary Medicine, Warsaw University of Life Sciences, Nowoursynowska 166, Warsaw, 02-776, Poland; 2 Vertebrates Morphology Department, University of Natural Sciences and Humanities, Siedlce, 08-110, Poland

**Keywords:** Horse, Proximal phalanx, Bone parameters, Computed tomography

## Abstract

**Background:**

Proximal phalanges in horses are among bones that are most prone to injuries. So far, the detailed analysis of densitometric and geometric parameters of both front legs proximal phalanges in horses has not been investigated. The aim of this study was to compare the densitometric and geometric parameters between proximal phalanges in equine both front legs with the use of peripheral quantitative computed tomography (pQCT).

**Methods:**

The study material comprised isolated both front legs proximal phalanges derived from 22 horses. The structure analysis of the proximal phalanges was conducted with the pQCT. The following bone parameters were determined: bone mineral content, volumetric bone mineral density, total bone area, trabecular area, cortical area, cortical thickness, periosteal circumference, endocortical circumference, Strength Strain Index. Tomographic analysis of proximal phalanges was conducted at three levels: at 15%, 50% and 85% of the bone length.

**Results:**

The statistical analysis showed that both the densitometric and geometric parameters of the bone at 50% and 85% of its length, did not present any statistically significant differences for the left or right proximal phalanges of the forelimb. At the same time, all examined parameters measured at 15% of the bone length, in the vicinity of the proximal metaphysis revealed significant statistical differences between both front legs proximal phalanges.

**Conclusions:**

The proximal phalanx parameters in the forelimbs are significantly different for the left and right proximal phalanx at 15% of the length and they indicate higher Strength Strain Index of the left bone in this location. The densitometric and geometric parameters of the bone at 50% and 85% of its length, did not present any statistically significant differences for the left or right proximal phalanges of the left and right forelimbs. The most serious changes caused by asymmetrical load of the thoracic limbs in horses occur near the proximal metaphysis, where the spongious substance is most abundant. This may happen because the metabolism of the spongious bone tissue is eight times faster compared to the compact bone tissue. Thus, any changes, including those caused by asymmetrical strain exerted on the right and left thoracic limbs, are the earliest to be observed.

## Background

Proximal phalanx fractures are one of the most common injuries that occur in the equine forelimb [1-4]. Based on four-year observations of 850 two-year old Thoroughbreds that underwent training, changes were found in as many as 19 out of 47 of all the limb bone fractures. Pastern bone fractures comprised 39.6% of all the fractures that were recorded in the 4-year-long observation period. Fractures of proximal navicular bone were slightly less frequent - 11 cases (22.9%) Fractures of other bones were rare [1]. Proximal phalanges are most often subject to longitudinal fractures. Such fractures are probably facilitated by the characteristic shape of the proximal bone end. On the articular surface of their proximal end, there is an the sagittal groove of the proximal phalanx with the sagittal ridge of the third metacarpal bone. The sagittal ridge acts as a wedge by “squeezing into” the cup-shaped depression, which can facilitate this type of fractures. It is also suggested that the decrease in the bone tissue parameters in the vicinity of proximal metaphysic of the proximal phalanx can be conducive to this type of fractures. Bone fractures are more likely to occur in the context of low bone tissue parameters such as decrease in the number of bone trabeculae, their volume, density and width [2,3]. Unfortunately, there are few reports on the bone tissue parameters of the proximal phalanges. The analysis comprises only densitometric parameters of the proximal phalanges [5]. However, the mechanic strength of the bone tissue, which affects its supporting functions, is affected not only by the mineral composition but also its spatial architecture [6-8]. Unfortunately, the evaluation of bone tissue quality is most often based on densitometric methods. The reason for the imperfections of these methods is the inability to evaluate the spatial structure of the bone trabeculae. The mineralization level is not the only factor affecting bone durability as the bone trabeculae architecture is also very important [8,9]. Computed Tomography (CT) allows for studying the geometry of the examined bone, providing important information regarding the bone microarchitecture [10].

The asymmetry of long bones has been examined in humans. The studies on this subject explicitly showed that there is a statistically significant difference between the bilateral thoracic limbs in humans. It was proved that the densitometric parameters in the studied bones are tightly related to hand preference [11-13]. Importantly, it was also shown that densitometric parameters of the bilateral femoral bones in humans are linked to handedness [14].

Particular feature of highly developed animals is the fact that they do not move in straight position, but tend to twist their body into one side. It can be said that they have a left-side or right-side tendency [15,16]. The right-sided have a natural tendency to move left, therefore in the open space most people and animals come full circles left. Right-sidedness is observed only in highly-developed mammals, including horses [17]. It is manifested while starting galloping with so called left leg more willingly, while the left thoracic limb is the leading one, so it is put forward first during horse’s action while running. A right-sided horse has a natural tendency to ‘break’ left, which means that when a horse gets scared of something, it usually nips out left. It was also observed that facing an obstacle, horses more frequently break left in front of it [17].

The fact the unequal loading inspired a lot of scientists and doctors to examine the influence of the unequal loading, not only on shaping but also on exposure to injuries of particular structures of the locomotor’s system of the bilateral limbs.

Comparison of the bilateral long bones of limbs in horses was conducted by morphometric methods. The studies confirmed the phenomenon of asymmetry regarding the femoral bones in this species [18] and the equine third metacarpal [19]. Importantly recent study found that the majority of proximal phalanx fractures in horses occur in the right leg [20].

In contrast to human medicine, where the pQCT method is becoming more frequently used [21-23] it is rarely applied in horses. It is related with species specificity, e.g. difficulty with keeping the animal still for several minutes, dimensions etc. [24].

Unfortunately, even tests on isolated equine bones with the use of the pQCT method are performed very rarely. So far, the above method has been used on radius and tibia [24,25], third metacarpal bone [26] and the distal sesamoid bone [27]. According to the authors’ knowledge, there is still insufficient number of research studies on comparative analysis of both densitometric and geometric parameters of the both front legs proximal phalanges in horses with the use of pQCT.

There is still lack of writing touching upon the issue of influence of the increased loading of one side of horse body on shaping of microstructure of long bones in bilateral limbs. The umber of studies that thoroughly compare densitometric and geometric parameters between bilateral ones of limbs in this species are still insufficient.

Due to the lack of studies that focus on how increased one-side load affects the microstructure of long bones in bilateral limbs and their densitometric features, studies that do address the issue are very important because of their cognitive value. Moreover, research on the possible influence of asymmetrical load on the pastern bone parameters may provide information on the nature of remodeling in the pastern bone tissue as a result of load.

The aim of this study was to compare the densitometric and geometric parameters between the both front legs proximal phalanges in the equine forelimb at 15%, 50% and 85% of bone length with the use of pQCT. It was also analyzed whether the right or left bone preference affected the values of the above parameters at the three measurement levels.

## Methods

### Horses

The material consisted of 44 proximal phalages from the front legs of 22 horses aged 2.5-15 (Table 1). There were no animals in which any anomalies concerning the pastern bones had been stated intravitally, and their slaughtering or death were not the result of any diseases. Before slaughter, or during visual inspection in the case of dead animals, morphological description was prepared based on the constitutional type of the horses. Each horse, according to its physical constitution, represented one of the following types: Warmblood horses (dolichomorphic type or light) and Coldblood horses (brachymorphic type or heavy) [28]. Warmblood horses were represented by Polish Halfbred Horse (warmblood breed crossed with thoroughbreds and used for sports purposes) (body weight of the adult horses ranged from 450 to 550 kg) n = 9. Coldblood horses on the other hand, comprised the animals from private farms in the Mazovia region - Polish Coldblood Horses, which are mainly used as draft animals (body weight of the adult horses ranged from 600 to 750 kg), n = 13.

**Table 1:** Age, sex, morphological type, use and reason for euthanasia in 22 used for Xtreme CT of the proximal phalanx

**Case number**	**Age in years**	**Sex**	**Morphological type: Polish Coldblood Horse (coldblood-heavy)-H; Polish Halfbred Horse (warmblood-light)-L**	**Use**	**Reason for euthanasia or slaughtering house**
1.	7	stallion	L	jumper	Bone fracture
2.	7	gelding	L	pleasure	Ruptured tendon
3.	4	gelding	H	farm	Slaughtering house
4.	15	gelding	H	farm	Slaughtering house
5.	6	mare	H	farm	Heart disease
6.	4	mare	L	jumper	Ruptured tendon
7.	7	gelding	L	pleasure	Kidney disease
8.	15	mare	H	farm	Metritis
9.	4	gelding	H	farm	Colic
10.	7	mare	L	pleasure	Colic
11.	4	mare	L	pleasure	Ruptured tendon
12.	4	mare	L	pleasure	Bone fracture
13.	12	gelding	H	farm	Slaughtering house
14.	2,5	stallion	L	pleasure	Ruptured tendon
15.	7	mare	H	farm	Slaughtering house
16.	8	gelding	H	farm	Slaughtering house
17.	8	mare	L	pleasure	Metritis
18.	5	mare	H	farm	Colic
19.	7	mare	H	farm	Slaughtering house
20.	4	stallion	H	farm	Ruptured tendon
21.	5	gelding	H	farm	Slaughtering house
22.	5	gelding	H	farm	Slaughtering house

Following the visual inspection, autopodia in thoracic limbs were dissected and in order to isolate the pastern bones from the soft tissues. After isolating, the bones were marked, placed in air-tight plastic bags and then kept 4 months in the temperature of −20°C.

### Computed tomography

The use of high resolution pQCT XCT Research SA Plus (Stratec Medizintechnik GmbH, Pforzheim Germany) allowed for analyzing the densitometric and geometric parameters of the proximal phalanges in horses. The following densitometric parameters were determined: BMC - bone mineral content per 1 mm slice in mg/mm and vBMD - volumetric bone mineral density in mg/cm³, the mean density of the total bone and geometrical parameters:

TOT_A - total bone area in mm^2;^ cross sectional area of the bone, after the soft tissue has been peeled off,

TRAB_A - trabecular area in mm^2^; cross sectional area of the trabecular area after the cortical and subcortical area has been peeled off,

CRT_A cortical area in mm^2^, the area that is assigned to be pure cortical, CRT_THK_C - the mean cortical thickness in mm,

PERI C - periosteal circumference in mm,

ENDO_C - endocortical circumference in mm,

SSI = RP_CM_W - Strength Strain Index - moment of resistance in mm³.

Tomographic analysis was performed at 15%, 50% and 85% of the bone length. The study was performed at the voxel size of 0.07 mm³ and scanning speed of 4 mm/min. The analyzed areas were determined by initial scanning (20 mm/s) and after morphometric measurements of the proximal phalanges. The threshold coefficient, which differentiates the compact bone from the cancellous bone was set at 0.900 cm^-1^. The thickness of the analysed slices was 0,07 mm;

Measurement areas of every bone were determined with the use of digital slide caliper of the length of every analysed bone. Next, the obtained results were entered in the computed tomography scanner software. After preliminary scanning, it was possible to set the reference line, tangent to the joint surface. Since the bone length had already been entered, now only the measurement lines in the selected bone area had to be added. In our study, those were the lines at 15%, 50% and 85% of the bone length.

## Statistical analysis

The analysis was performed separately for the previously described three measurement levels for two groups of proximal phalanges - left and right. The first step of the statistical analysis was to verify the compliance with the normal distribution. For the features, which were not compliant with the normal distribution, the differences between the left and right proximal phalanges were tested with a non-parametric test - Wilcoxon paired samples (Z) p ≤ 0.05. For the remaining features, whose distribution was compliant with normal distribution, the differences between the bilateral bones were tested with the t-Student test (t) for combined variables at p ≤ 0.05.

The densitometric and geometric features in the bilateral proximal phalanges (left and right in thoracic limb) in horses at 50% of the bone length and at 15% and 85% of the bone length were described and compared. The differences between parameters for the both front legs proximal phalanges, depending on the measurement location, were presented in tables and on box plots. The whiskers in boxes indicate the minimal and maximal values of a given feature. The box size shows the variability in 50% of the specimen (threshold of 25-75%). Additionally, the graph shows the average value. All calculations were performed using the Statistica 9.0 software (StatSoft, Inc. Tulsa, USA), at p ≤ 0.05.

## Results

The densitometric and geometric parameters of the both front legs proximal phalanges in horses were determined by pQCT at 15%, 50% and 85% of the bone length. The results comparing the parameters of the bilateral proximal phalanges on three examined sections are illustrated in Table 2. The statistical analysis showed that both the densitometric and geometric parameters of the bone at 50% and 85% of its length, did not present any statistically significant differences for the left or right proximal phalanges of forelimbs. It is confirmed by the values of t-Student test (t) and the Wilcoxon paired samples (Z) presented in Table 2. The t-Student values for the dependent samples prove that there is no difference between the left and right forelimbs at 50% of the bone shaft as far as the investigated features are concerned. The values of probability are >0.05. The probability values indicate that the null hypotheses assuming lack of differences between the left and the right limbs were taken as acceptable at the probability of 95%.

**Table 2:** The value of t-student test (t) and the order of Wilcoxon paired samples (Z) testing the difference between the pastern bones of the left and right forelimbs in 22 horses

**The value of tests**			
Bone parameter	15% slice	50% slice	85% slice
BMC mg/mm	Z = 3.977**	Z = 0.081	t = 0.348
	p = 0.000	p = 0.935	p = 0.730
vBMD mg/cm³	Z = 3.782**	Z = 0.828	Z = 0.666
	p = 0.000	p = 0,407	p = 0.505
TOT_A mm^2^	Z = 4.12**	t = − 0.527	t = − 0,735
	p = 0.000	p = 0,603	p = 0.470
TRAB_A mm^2^	Z = 4.107**	t = − 0.55	t = − 0.723
	p = 0.000	p = 0,588	p = 0.477
CRT_A mm^2^	t = − 2.165*	t = 1.246	Z = 0.016
	p = 0.002	p = 0,226	p = 0,987
CRT_THK_C mm	t = 4.42**	Z = 1.315	t = 0.999
	p = 0.000	p = 0,188	p = 0,328
PERI C mm	Z = 4.12**	t = − 0.416	Z = 0.146
	p = 0.000	p = 0,681	p = 0,883
ENDO_C mm	t = − 12.468**	t = − 1.013	t = − 0.948
	p = 0.000	p = 0,332	p = 0,354
RP_CM_W mm³	Z = 3.945**	Z = 0.535	t = 0.00
	p = 0.000	p = 0,592	p = 1,000

*significant statistical differences at P ≤ 0.05.

** highly significant statistical differences at P ≤0.01.

Detailed values for the described densitometric and geometric parameters of the both front legs proximal phalanges in horses examined at 15%, 50% and 85% of the bone length were presented in Tables 3, 4, 5.

**Table 3:** Descriptive statistics for 22 pairs of pastern bones examined at 15% of the bone shaft

**Bone**	**Right proximal phalanx**				**Left proximal phalanx**			
**parameter**	**Minimum**	**Maximum**	**Mean**	**SD**	**Minimum**	**Maximum**	**Mean**	**SD**
BMC mg/mm	488	932	764	105	635	1305	1041	182
vBMD mg/cm³	538	820	713	90	454	669	554	60
CRT_THK_C mm	4	8	6	1	4	7	5	1
RP_CM_W mm³	4145	10027	7630	1658	4784	21246	13160	4518
TOT_A mm^2^	816	1371	1080	153	1113	2496	1894	351
TRAB_A mm^2^	367	617	486	69	501	1121	852	158
CRT_A mm^2^	404	737	609	85	408	903	681	148
PERI C mm	101	131	116	8	118	177	154	15
ENDO_C mm	58	99	76	11	94	149	123	15

**Table 4:** Descriptive statistics for 22 pairs of pastern bones examined in the middle of the bone shaft (50% of the bone length)

**Bone**	**Right proximal phalanx**				**Left proximal phalanx**			
**parameter**	**Minimum**	**Maximum**	**Mean**	**SD**	**Minimum**	**Maximum**	**Mean**	**SD**
BMC mg/mm	482	874	744	99	470	857	730	111
vBMD mg/cm³	560	823	741	68	538	820	722	84
CRT_THK_C mm	5	8	7	1	4	8	6	1
RP_CM_W mm³	2999	10218	7132	1783	2937	10027	7053	1929
TOT_A mm^2^	585	1255	1013	163	592	1372	1024	195
TRAB_A mm^2^	263	565	455	73	266	617	461	88
CRT_A mm^2^	427	707	601	78	397	695	585	83
PERI C mm	86	126	112	9	86	131	113	11
ENDO_C mm	45	96	71	11	50	99	73	13

**Table 5:** Descriptive statistics for 22 pairs of pastern bones examined at 85% of the bone shaft

**Bone**	**Right proximal phalanx**				**Left proximal phalanx**			
**parameter**	**Minimum**	**Maximum**	**Mean**	**SD**	**Minimum**	**Maximum**	**Mean**	**SD**
BMC mg/mm	550	991	819	113	544	980	813	119
vBMD mg/cm³	530	776	679	62	430	760	661	83
CRT_THK_C mm	4	7	6	1	2	7	6	1
RP_CM_W mm³	4225	12123	9032	2256	2775	12615	8883	2432
TOT_A mm^2^	759	1567	1217	206	830	1725	1246	220
TRAB_A mm^2^	341	703	547	93	373	776	560	99
CRT_A mm^2^	441	726	603	85	244	707	590	107
PERI C mm	98	140	123	11	102	147	125	11
ENDO_C mm	63	113	87	13	67	120	120	15

**Figure 1:**
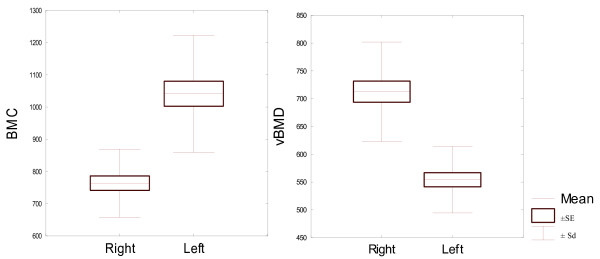
**Densitometric parameters for 22 pairs of proximal phalanges analysed in 15% of the bone length. **Left - BMC mg/mm, right - vBMD mg/cm3.

**Figure 2:**
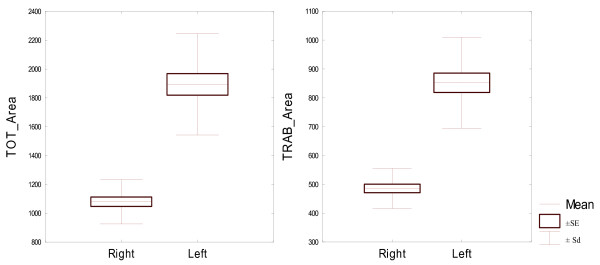
**Geometric parameters, describing bone surface in the analysed area for 22 pairs of proximal phalanges analysed in 15% of the bone length. **Left - TOT_A mm^2^, right - TRAB_A mm^2^.

**Figure 3:**
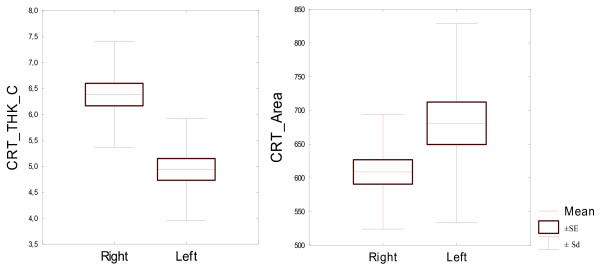
**Geometric parameters for the compact bone tissue in the analysed area for 22 pairs of proximal phalanges analysed in 15% of the bone length.** Left - CRT_THK_C mm, right CRT_A mm^2^.

**Figure 4:**
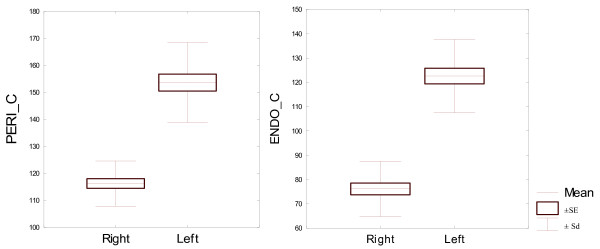
**Geometric parameters for the external (subperiosteal) and internal circumferences of the compact bone in the analysed area for 22 pairs of proximal phalanges analysed in 15% of the bone length. **Left PERI C mm, right ENDO C mm CRT_THK_C.

**Figure 5:**
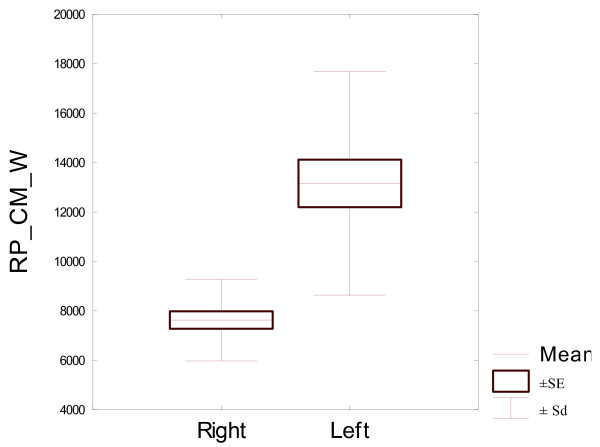
**Expected RP_CM_W mm³, for 22 pairs of proximal phalanges analysed in 15% of the bone length.** RP_CM_W.

All bone parameters measured at 15% of the bone length proved to be statistically significantly different between the left and right proximal phalanges (Table 5). Both the densitometric parameters measured at 15% of the bone length proved to be significantly different between the left and right proximal phalanges. And so the bone mineral content was significantly higher in the left bone than in the right bone. Volumetric bone mineral density in this level was significantly higher in the right bone. Both the bone geometric parameters, describing bone surface in the analysed area, recorded in 15% of the bone length, were also significantly different for bilateral proximal phalanges. Average analysed total bone area was larger for the left proximal phalanges. Trabecular area analysed was also significantly larger in the left proximal phalanx, compared the right proximal phalanx. Differences between geometric parameters for the compact bone tissue in 15% of the bone length were also statistically significant for the both front legs proximal phalanges. Cortical thickness was larger in right bone but cortical area was larger in the left one. This was due to the fact that left proximal phalanges displayed significantly higher periosteal circumference and endocortical circumference, and so significantly larger diameter, which resulted in a larger cortical area, even though cortical thickness was smaller. Periosteal circumference and endocortical circumference of the left side bones was significantly higher. Expected Strength Strain Index - SSI was significantly higher in the left proximal phalanx than in the right one. Due to the fact that all bone parameters measured at 15% of the bone length proved to be statistically significantly different in the left and right proximal phalanges (Table 2), they were described in detail and illustrated in Figures 1, 2, 3, 4, 5.

## Discussion

Comparative analyses of bilateral limb bones, conducted with the use of the pQCT method, have been performed on the radius and tibia at 50% and 80% of the bone length. The conducted research did not show statistically significant differences in the analysed densitometric and geometric parameters between bilateral radius and tibia of horses at both studied levels [24]. Research on the same bones was also carried out at the levels from 15% to 95% (every 10%) of the bone length. They confirmed the earlier observations of no statistically significant differences between the analysed densitometric and geometric parameters for bilateral radius and tibia.

Within the present research, measurements of bilateral proximal phalanges in horses have been taken at three levels: 15%, 50% and 85% of the bone length. Other researchers have also conducted studies on the both front legs proximal phalanges in horses. However, they compared only densitometric parameters as the analysis was carried out by dual energy x-ray absorptiometry (DXA). When using the above method, the authors of the present paper have not observed any statistically significant differences regarding bone mineral density and bone mineral content between bilateral proximal phalanges [5].

We analysed densitometric as well as geometric parameters for the both front legs proximal phalanges in horses. With the use of pQCT, we demonstrated that all the parameters studied, both densitometric and geometric, measured at 50% and 85% of the bone length, did not present any statistically significant differences between the right and left proximal phalanges of the front limb. Thus, the results correspond to the observations regarding other long bones [24,25]. Interestingly, at the same time, our research proved that all the bone parameters mentioned above, measured at 15% of the bone length, were significantly different for the right and left proximal phalanges. In our research, volumetric bone mineral density at 15% of the bone length was significantly higher in the right proximal phalanx, whereas geometrical parameters and bone strength (SSI) in this area were significantly higher in left pastern bones. As it turns out, considerably higher increase in geometrical parameters of left pastern bones near the proximal metaphysis, compared to right pastern bones, outweighed the relatively small increase in mineral nutrients. This resulted in lower vBMD in left pastern bones, compared to right pastern bones. Tests conducted on the radius and tibia of horses revealed that the vBMD value did not increase as a result of physical exercise but bone strength to forces did. Physical exercise, on the other hand, caused an increase of bone mineral content in the radius and tibia [24]. Our research indicated that BMC measured at 15% of the proximal phalanx length was significantly higher in the left proximal phalanx.

Both parameters describing the analysed bone areas: total bone area and trabecular area, were also significantly higher in left proximal phalanx. Moreover, left side bones at 15% of the bone length presented higher periosteal circumference and endocortical circumference, parameters whose value grew with the increase in bone strength. Bone section plays an important role in their resistance to bending and twisting. Larger diameter of left pastern bones proves they are more resistant to these forces. The above parameters are, for example, significantly higher in horses that are trained, compared to those that do not undergo training [25].

According to other researchers’ observations, forces induced by physical exercise such as cantering and galloping cause an increase in periosteal circumference and endocortical circumference as well as Strength Strain Index in the radius and tibia. On the other hand, there was no increase in cortical thickness in trained horses. Values of this parameter did not present significant differences between the trained and untrained groups of horses [25].

Therefore, it can be assumed that the higher cortical thickness of the right side bones that was observed in our study does not translate into their higher strength.

At the same time, our study showed that another parameter describing the compact bone tissue, cortical area, at 15% of the bone length was significantly higher in left proximal phalanges. Higher strength of left side pastern bones compared to right pastern bones at 15% of the bone length is also evidenced by the fact that the expected Strength Strain Index in this area was significantly higher in the left proximal phalanx. Combined effect of the increase in such parameters as periosteal circumference, endocortical circumference and cortical area signifies the rise in bone strength [25,29]. Our study clearly showed that the parameters in bilateral proximal phalanges differ significantly at 15% of the bone length. It was also observed that all the parameters that suggest higher strength of bones to such forces as: bone mineral content, total bone area, trabecular area, cortical area, periosteal circumference, endocortical circumference, Strength Strain Index, were significantly higher for left proximal phalanges. Strength Strain Index was even two times higher.

Bones undergo constant remodeling during lifespan. Bone cell activity is determined by general factors such as hormones and vitamins, and local factors, e.g. Insuline -like Growth Factor, interleukins (IL-6, IL-3, IL-1), or TGFβ -Transforming Growth Factor [30]. For the remodeling process to begin, i.e. for the bones to change their structure, initiating factors for osteoblast precursors are necessary [31]. What is also important with regard to remodeling is physical factors, which are particularly significant in determining the microstructure of the bone tissue [32-34]. Due to the fact that our study involved comparing bilateral bones in the same animal, we were able to assess the influence of asymmetrical strain exerted during the animal’s movement on the differentiation of geometrical and densitometric parameters at given lengths of the pastern bone. Since the study showed statistically significant differences at 15% of the bone length, which is quite novel, it is recommended to conduct a similar study in a larger population of horses.

## Conclusions

Our results confirm the observation that every bone reacts individually to load forces, which is related to its location, shape and function. Simultaneously, mechanisms that modulate bone density and microstructure can cause significant changes in the bone at a certain level, leaving the remaining bone length unaffected. Proximal phalanges undergo the most substantial changes in microstructure in the vicinity of proximal metaphysis. Another observation is that, compared to right side proximal phalanges, left side proximal phalanges in forelimbs in the vicinity of the proximal metaphysis are characterised with significantly higher parameters, which manifest higher Strength Strain Index.

In our study, the most distinct differences between the right and left pastern bones were observed at 15% of the diaphyseal length, i.e. in the areas where the spongious substance is most abundant. According to our hypothesis, this may happen because the metabolism of the spongious bone tissue is eight times faster compared to the compact bone tissue. Bone metabolism is most intensive in the spongious substance due to a larger area for osteoblasts and osteoklasts to operate, larger blood supply and better access to tissue fluids. Thus, the spongious substance displays the highest diagnostic value since any pathological changes as well as changes caused by different strains are the earliest to be observed here. Our observations suggest that this type of research should be continued within a larger population.

## Abbreviations

pQCT, Peripheral quantitative computed tomography; BMC, Bone mineral content; vBMD, Volumetric bone mineral density; TOT_A, Total bone area; TRAB_A, Trabecular area; CRT_A, Cortical area; CRT_THK_C, The mean cortical thickness; PERI C, Periosteal circumference; ENDO_C, Endocortical circumference; SSI = RP_CM_W, Strength Strain Index; t, t-student test; Z, Wilcoxon paired samples test; SD, Standard deviation; P, Significant statistical differences..

## Competing interests

The authors declare that they have no competing interests.

## Authors’ contributions

MD - the initiator of the study, who conducted the main experimental part, performed the statistical calculations and described the results. AC - result advisor, who participated in collecting the research material. Both authors read and approved the final manuscript.
